# When the mean is an empty plate for clinicians and scientists, and a full plate for politicians and writers

**DOI:** 10.1590/2176-9451.19.5.014-015.edt

**Published:** 2014

*"Statistics. The science that says if I ate a whole chicken and you didn't eat any,* *then each of us ate half a chicken."* Dino Segrè, Italian writer 

Orthodontics, as well as other branches of science, completely adores arithmetic mean. We can routinely go on stating that orthodontic treatment lasts for 24 months by using mean to speak the truth. However, in some cases, this parameter should not be assessed in isolation or not even employed at all.

The mean is a measure of central tendency near of which most data are gathered. Nevertheless, using it to state what happens with most patients requires compliance with a few requirements. Should they be absent, we go on employing the mean to describe clinical as well as scientific outcomes. A happy illusion caused by habit.

Mean will be of little importance and will produce scanty information whenever there is great variability in whatever is being assessed. And variability is routine in the field of biology. 

Suppose you are assessing patients' treatment time. We have the premise that, on average, orthodontic treatment lasts for 24 months. Nevertheless, should you assess each patient's register, you will find a reasonable number of cases finished within 12 months and many others finished after 36 months. 

Should that be the case, the mean is an inaccurate measure that will probably lead to mistakes if you insist in using it to predict your next patient's treatment time. Any experienced orthodontist - and many inexperienced as well - has already learned this concept. In these cases, variability is a much more interesting measure than the mean. Investigations, whether clinical or scientific, should dwell on the reasons for such variation, not on a measure (the mean) including a small minority.

**Figure 1:**
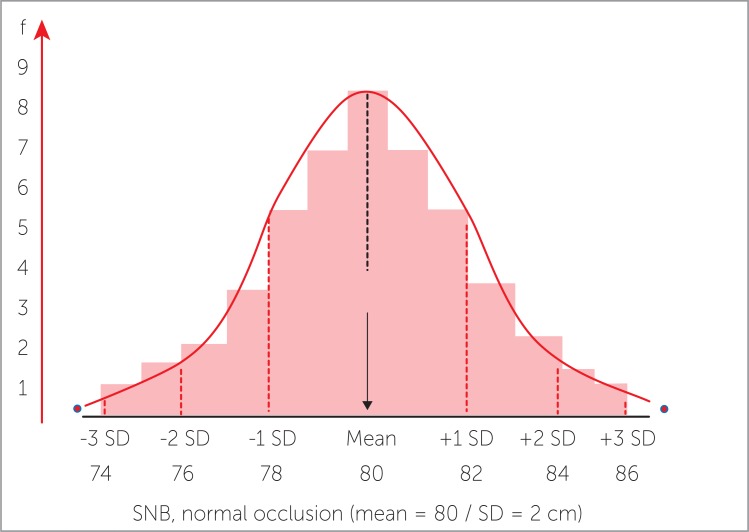
SNB angle of a given population with normal occlusion (fictitious data). Symmetrical distribution suggests normality of the curve in which the mean is the value most frequently found. The further a value is from the mean, the lower the chances of being identified

The mean is misused, for instance, when we assess asymmetrical distribution, also known as abnormal distribution. An example of such a case is reading the SNB angle of a given population with normal occlusion, as fictitiously illustrated in Figure 1. The mean (80^o)^ will be the value most frequently observed, and the further we are from this value - adjusted upward or downward, symmetrically - the lower the chances of finding the reference value. Thus, in a normal sample, we are more likely to find a SNB value of 78^o^ than 74^o^, for example. Therefore, in normal distribution, the mean is the value most frequently found. Moreover, the further we are from the mean, the lower the chances of finding a given event, adjusted upward or downward.

**Figure 2:**
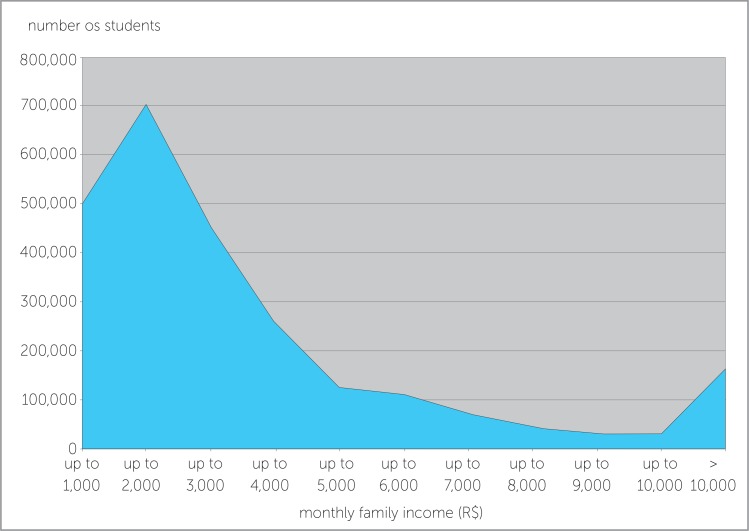
Monthly income (in R$, Brazilian Reais) of families with higher education students (Source: IBGE,1 1998). Note evident asymmetry of the curve. The mean, which is the value most frequently found, is skewed to the right (up to R$ 2,000.00 - US$ 802.18). Most families have a monthly income of R$ 3,000.00 (US$ 1,203.27).

This premise is broken in cases of major asymmetries in data distribution. Figure 2 shows the income distribution of Brazilian families with higher education students.^1^ The value most frequently found is an income of two thousand Reais (US$ 802,18) - too little in terms of family income. The mean, however, is skewed to the right due to a small number of families with a higher income. This limited number of families with a much higher income will significantly increase the mean value. Thus, the mean value will be considerably above two thousand Reais (US$ 802.18) - something around five thousand Reais (US$ 2,005.45), which seems a lot better.

In the aforementioned example, using the mean is a mistake, given that it will overestimate the central tendency of data. During these times of election, this practice would be useful to manipulate data by means of choosing the wrong central measure to describe a problem. And it seems to be a common and intentional practice in politics aimed at gaining advantage or expressing criticism. Misusing the mean, however, is not common only in politics, but also in science and literature. In arts, however, mistakes might have an ironic connotation, as in the aforementioned epigraph.

In science, the bottom of the issue is deeper: on average, six feet under - but with some degree of variability, depending on the size of the foot.

David Normando - editor-in-chief (davidnormando@hotmail.com)
